# Supplementary material for: Deforestation intensifies daily temperature variability in the northern extratropics
Source: Nat Commun. 2022 Oct 10;13:5955. doi: 10.1038/s41467-022-33622-0 (PMC9550804; doi:10.1038/s41467-022-33622-0)
Supplement: Supplementary file 1 — Supplementary Information [file 41467_2022_33622_MOESM1_ESM.docx]

Supplementary Information for

**Deforestation intensifies daily temperature variability in the northern extratropics**

Jun Ge^*1, 2^, Qi Liu^1, 2^, Beilei Zan^3, 4, 5^, Zhiqiang Lin^6^, Sha Lu^1, 2^, Bo Qiu^1, 2^ and Weidong Guo^*1, 2^

^1^School of Atmospheric Sciences, Nanjing University, Nanjing, China.

^2^Joint International Research Laboratory of Atmospheric and Earth System Sciences, Nanjing University, Nanjing, China.

^3^Collaborative Innovation Center on Forecast and Evaluation of Meteorological Disasters/Key Laboratory of Meteorological Disaster, Nanjing University of Information Science and Technology, Nanjing, China.

^4^Ministry of Education/International Joint Research Laboratory on Climate and Environment Change, Nanjing University of Information Science and Technology, Nanjing, China.

^5^School of Atmospheric Sciences, Nanjing University of Information Science and Technology, Nanjing, China.

^6^School of Atmospheric Sciences, Chengdu University of Information Technology, Chengdu, China.

These authors contributed equally: Jun Ge and Qi Liu

Corresponding author: Jun Ge ([junge@nju.edu.cn](mailto:junge@nju.edu.cn)) and Weidong Guo ([guowd@nju.edu.cn](mailto:guowd@nju.edu.cn))

**Contents of this file**

Supplementary Table 1 to 3

Supplementary Figures 1 to 8

**Supplementary Table 1** The simulations and models used in this study. The variant label denotes the member used; otherwise, the model is not used.

|  | piControl | deforest-globe | historical | hist-noLu | hist-nat | ssp370 | ssp370-ssp126Lu |
| --- | --- | --- | --- | --- | --- | --- | --- |
| ACCESS-ESM1-5^1^ |  |  | r1i1p1f1 | r1i1p1f1 | r1i1p1f1 | r1i1p1f1 | r1i1p1f1 |
| BCC-CSM2-MR^2^ |  |  | r1i1p1f1 | r1i1p1f1 | r1i1p1f1 | r1i1p1f1 | r1i1p1f1 |
| CanESM5^3^ | r1i1p2f1 | r1i1p2f1 |  |  |  |  |  |
| CESM2^4^ |  |  | r1i1p1f1 | r1i1p1f1 | r1i1p1f1 |  |  |
| CMCC-ESM2^5^ | r1i1p1f1 | r1i1p1f1 | r1i1p1f1 | r1i1p1f1 |  | r1i1p1f1 | r1i1p1f1 |
| CNRM-ESM2-1^6^ | r1i1p1f2 | r1i1p1f2 | r1i1p1f2 | r1i1p1f2 |  | r1i1p1f1 | r1i1p1f1 |
| EC-Earth3-Veg^7^ | r1i1p1f1 | r1i1p1f1 | r1i1p1f1 | r1i1p1f1 |  |  |  |
| GFDL-ESM4^8^ |  |  | r1i1p1f1 | r1i1p1f1 | r1i1p1f1 |  |  |
| MPI-ESM1-2-LR^9^ |  |  | r1i1p1f1 | r1i1p1f1 |  | r1i1p1f1 | r1i1p1f1 |
| UKESM1-0-LL^10^ | r1i1p1f2 | r1i1p1f2 | r1i1p1f2 | r1i1p1f2 |  | r1i1p1f1 | r1i1p1f1 |

**Supplementary Table 2** The atmospheric variables used in this study and the corresponding model output names.

| Atmospheric variables | Model output names |
| --- | --- |
| 2-meter temperature | tas |
| Maximum 2-meter temperature | tasmax |
| Minimum 2-meter temperature | tasmin |
| 10-meter wind speed | uas, vas |
| Surface downward shortwave radiation | rsds |
| Surface upward shortwave radiation | rsus |
| Surface net shortwave radiation | rss |
| Surface downward longwave radiation | rlds |
| Surface upward longwave radiation | rlus |
| Surface net longwave radiation | rls |
| Surface sensible heat flux | hfss |
| Surface latent heat flux | hfls |

**Supplementary Table 3** Detailed information on the paired sites used in this study. The second row gives the name of each site; the third and fourth rows give the latitude and longitude information of each site, respectively; the fifth row gives the land cover type of each site (CRO: croplands; GRA: grasslands; OSL: open shrublands; DBF: deciduous broadleaf forests; ENF: evergreen needleleaf forests); the sixth and seventh rows give the elevation difference and the horizontal distance between the forest and openland sites in each pair, respectively; the eighth row gives the time period that the observations from both the forest and openland sites are available for comparison.

| Pair ID | Name | Latitude (^o^N) | Longitude (^o^E) | Land cover | Elevation difference (m) | Distance (km) | Period |
| --- | --- | --- | --- | --- | --- | --- | --- |
| 1 | IT-CA2 | 42.3772 | 12.026 | CRO | 0 | 0.36 | 2011-2013 |
|  | IT-CA1 | 42.3804 | 12.0266 | DBF |  |  |  |
| 2 | IT-CA2 | 42.3772 | 12.026 | CRO | 3 | 0.44 | 2011-2013 |
|  | IT-CA3 | 42.38 | 12.0222 | DBF |  |  |  |
| 3 | IT-CA2 | 42.3772 | 12.026 | CRO | 40 | 8.75 | 2011-2012 |
|  | IT-Ro2 | 42.3903 | 11.9209 | DBF |  |  |  |
| 4 | FR-Gri | 48.8442 | 1.9519 | CRO | 35 | 73.3 | 2005-2013 |
|  | FR-Fon | 48.4764 | 2.7801 | DBF |  |  |  |
| 5 | BE-Lon | 50.5515 | 4.7461 | CRO | 326 | 92.82 | 2004-2014 |
|  | BE-Vie | 50.3050 | 5.9980 | DBF |  |  |  |
| 6 | US-Dk1 | 35.9712 | -79.0934 | GRA | 0 | 0.68 | 2003-2008 |
|  | US-Dk2 | 35.9736 | -79.1004 | DBF |  |  |  |
| 7 | US-Dk1 | 35.9712 | -79.0934 | GRA | 5 | 0.78 | 2004-2008 |
|  | US-Dk3 | 35.9782 | -79.0942 | ENF |  |  |  |
| 8 | CZ-BK1 | 49.4944 | 18.5429 | GRA | 20 | 0.96 | 2004-2006 |
|  | CZ-BK2 | 49.5021 | 18.5369 | ENF |  |  |  |
| 9 | DE-Gri | 50.9495 | 13.5125 | GRA | 5 | 4.12 | 2004-2010 |
|  | DE-Tha | 50.9636 | 13.5669 | ENF |  |  |  |
| 10 | DE-Gri | 50.9495 | 13.5125 | GRA | 350 | 23.49 | 2008-2010 |
|  | DE-Obe | 50.7836 | 13.7196 | ENF |  |  |  |
| 11 | NL-Hor | 52.2404 | 5.0713 | GRA | 22.8 | 46.55 | 2004-2011 |
|  | NL-Loo | 52.1666 | 5.7436 | ENF |  |  |  |
| 12 | US-NC1 | 35.8118 | -76.7119 | OSL | 0 | 4.03 | 2005-2009 |
|  | US-NC2 | 35.803 | -76.6685 | ENF |  |  |  |
| 13 | US-Wi6 | 46.6249 | -91.2982 | OSL | 40 | 15.27 | 2002-2003 |
|  | US-Wi3 | 46.6347 | -91.0987 | DBF |  |  |  |
| 14 | US-Wi6 | 46.6249 | -91.2982 | OSL | 19 | 16.22 | 2002-2003 |
|  | US-Wi4 | 46.7393 | -91.1663 | ENF |  |  |  |
| 15 | US-Wi6 | 46.6249 | -91.2982 | OSL | 22 | 16.57 | 2002 |
|  | US-Wi0 | 46.6188 | -91.0814 | ENF |  |  |  |
| 16 | CA-SF3 | 54.0916 | -106.0053 | OSL | 20 | 19.87 | 2001-2005 |
|  | CA-SF2 | 54.2539 | -105.8775 | ENF |  |  |  |
| 17 | CA-NS6 | 55.9167 | -98.9644 | OSL | 16 | 27.43 | 2001-2005 |
|  | CA-NS2 | 55.9058 | -98.5247 | ENF |  |  |  |
| 18 | CA-NS6 | 55.9167 | -98.9644 | OSL | 16 | 30.25 | 2001-2005 |
|  | CA-NS1 | 55.8792 | -98.4839 | ENF |  |  |  |
| 19 | CA-NS6 | 55.9167 | -98.9644 | OSL | 16 | 30.48 | 2001-2005 |
|  | CA-NS5 | 55.8631 | -98.485 | ENF |  |  |  |
| 20 | CA-NS6 | 55.9167 | -98.9644 | OSL | 16 | 36.29 | 2001-2005 |
|  | CA-NS3 | 55.9117 | -98.3822 | ENF |  |  |  |
| 21 | CA-SF3 | 54.0916 | -106.0053 | OSL | 4 | 45.41 | 2003-2006 |
|  | CA-SF1 | 54.485 | -105.8176 | ENF |  |  |  |
| 22 | DE-Kli | 50.8929 | 13.5225 | CRO | 100 | 8.46 | 2004-2014 |
|  | DE-Tha | 50.9636 | 13.5669 | ENF |  |  |  |
| 23 | DE-Kli | 50.8929 | 13.5225 | CRO | 255 | 18.42 | 2008-2014 |
|  | DE-Obe | 50.7836 | 13.7196 | ENF |  |  |  |
| 24 | DE-RuS | 50.8659 | 6.4472 | CRO | 390 | 69.96 | 2011-2014 |
|  | BE-Vie | 50.3050 | 5.9980 | DBF |  |  |  |


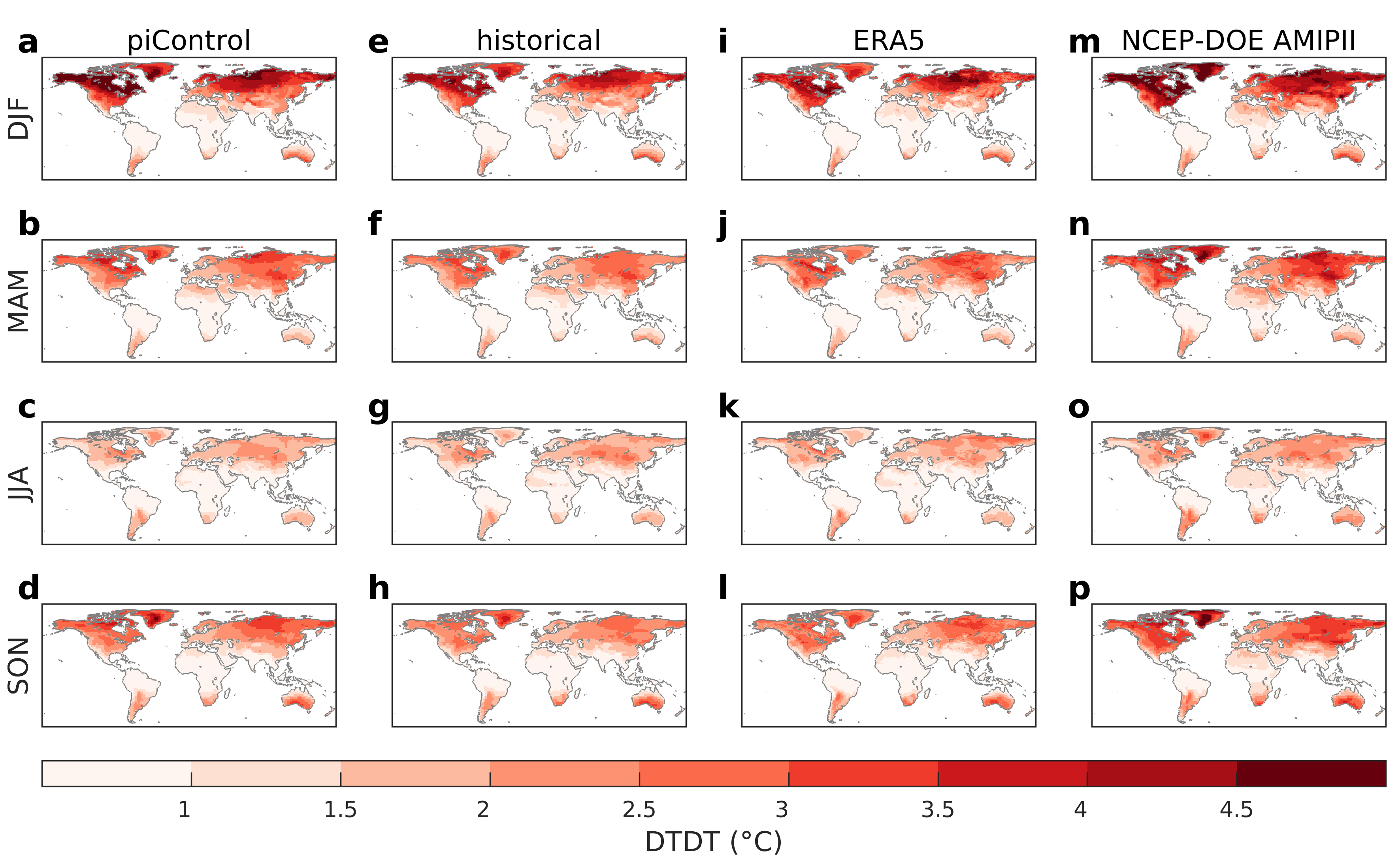


**Supplementary Fig. 1** **The multiyear mean day-to-day temperature variability (DTDT).** The mean DTDT from **a-d** the piControl simulation, **e-h** the historical simulation, **i-l** the ERA5 reanalysis and **m-p** the NCEP-DOE AMIP-II reanalysis in (**a**, **e**, **i**, **m**) DJF (December, January and February), (**b**, **f**, **j**, **n**) MAM (March, April and May), (**c**, **g**, **k**, **o**) JJA (June, July and August) and (**d**, **h**, **l**, **p**) SON (September, October and November). The DTDT values are averaged over the period of years 51-80 for piControl and 1985-2014 for historical and the two reanalysis datasets.


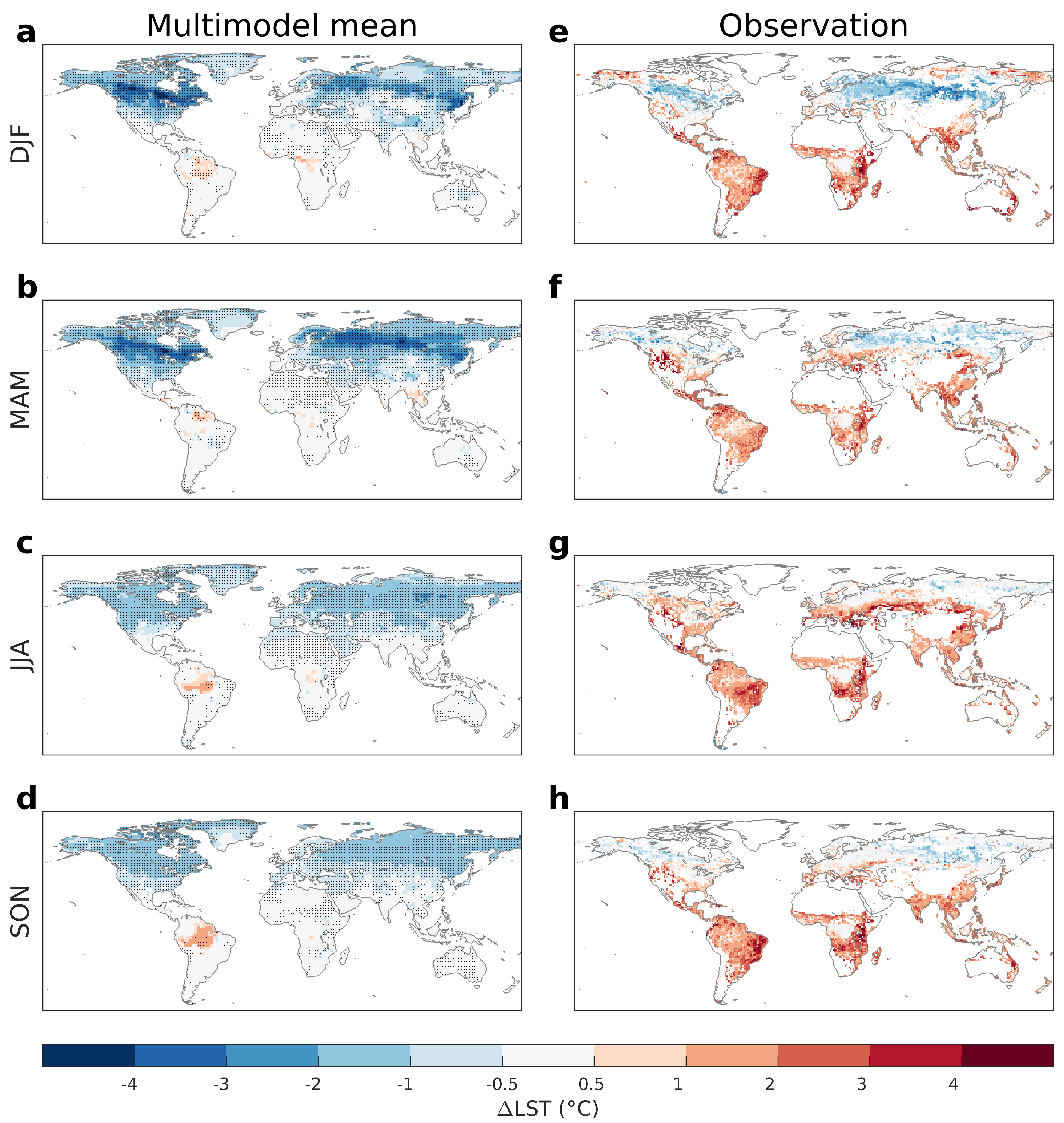


**Supplementary Fig. 2 The** **biogeophysical effect of deforestation on mean surface temperature.** The deforestation effect in (**a**, **e**) DJF (December, January and February), (**b**, **f**) MAM (March, April and May), (**c**, **g**) JJA (June, July and August) and (**d**, **h**) SON (September, October and November) obtained from (**a-d**) the simulations (deforest-globe minus piControl) and (**e-h**) satellite observations^11^. The black dots in **a**-**d** denote that all five models agree on the sign of the temperature response to deforestation.


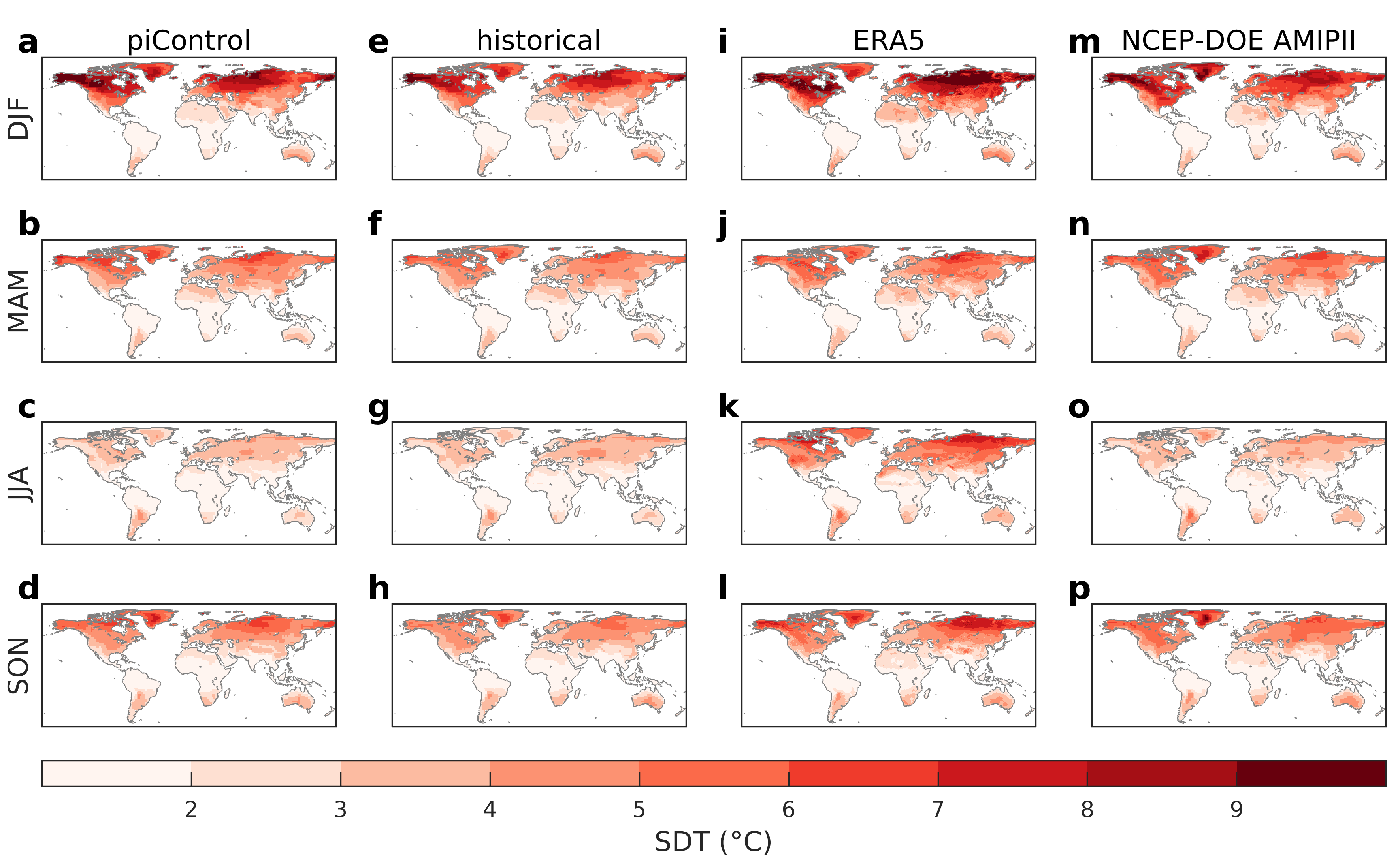


**Supplementary Fig. 3 The multiyear mean standard deviation of daily temperature (SDT).** The mean SDT from **a-d** the piControl simulation, **e-h** the historical simulation, **i-l** the ERA5 reanalysis and **m-p** the NCEP-DOE AMIP-II reanalysis in (**a**, **e**, **i**, **m**) DJF (December, January and February), (**b**, **f**, **j**, **n**) MAM (March, April and May), (**c**, **g**, **k**, **o**) JJA (June, July and August) and (**d**, **h**, **l**, **p**) SON (September, October and November). The SDT values are averaged over the period of years 51-80 for piControl and 1985-2014 for historical and the two reanalysis datasets.


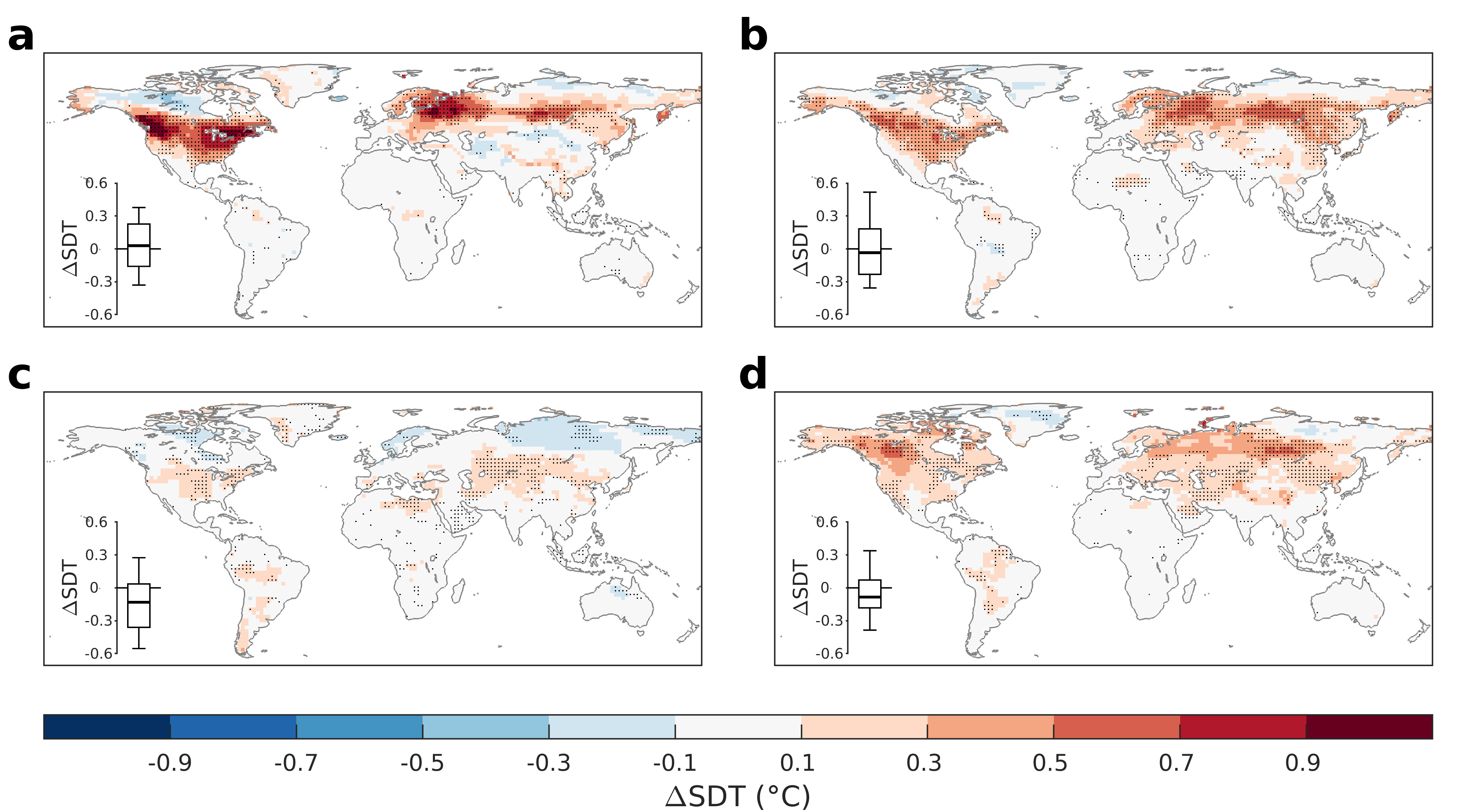


**Supplementary Fig. 4 The biogeophysical effect of deforestation on the standard deviation of daily temperature (SDT).** The multimodel mean effect of the idealized deforestation (deforest-globe minus piControl) on SDT in (**a**) DJF (December, January and February), (**b**) MAM (March, April and May), (**c**) JJA (June, July and August) and (**d**) SON (September, October and November). The black dots denote that all models agree on the sign of the SDT change. The box-and-whisker plot embedded in each panel shows the SDT differences between the paired forest and openland sites (openland minus forest; Fig. S5) from the FLUXNET and AmeriFlux datasets. The horizontal black line of the box denotes the median. The bottom and top edges of the box denote the 25^th^ and 75^th^ percentiles, respectively. The whiskers extend to the 10^th^ and 90^th^ percentiles.


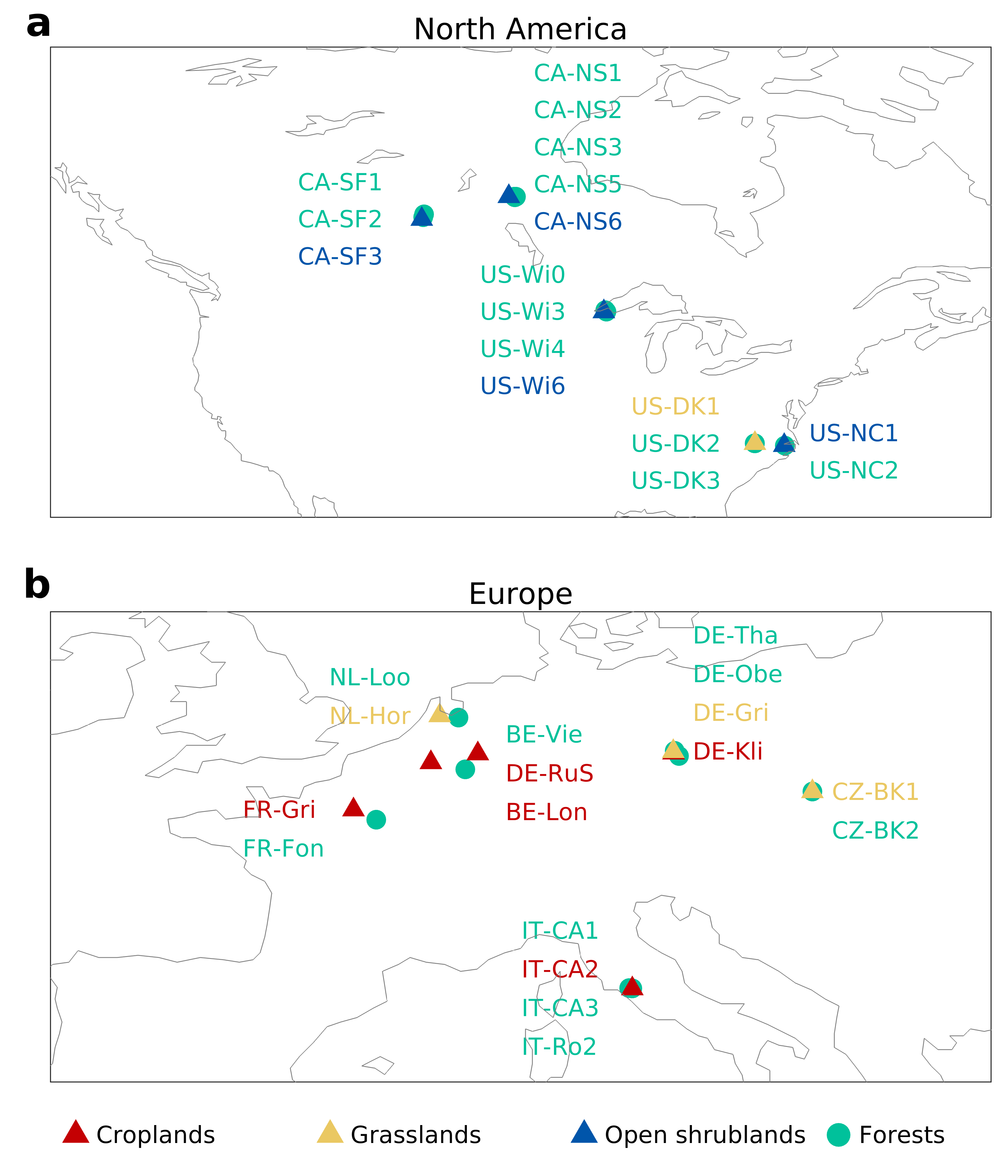


**Supplementary Fig. 5 Location of the paired sites.** Location of the paired forest and openland sites in (**a**) North America and (**b**) Europe. The forest sites are denoted by the green color. The grassland, cropland and open shrubland sites are denoted by the blue, red and yellow circles, respectively.


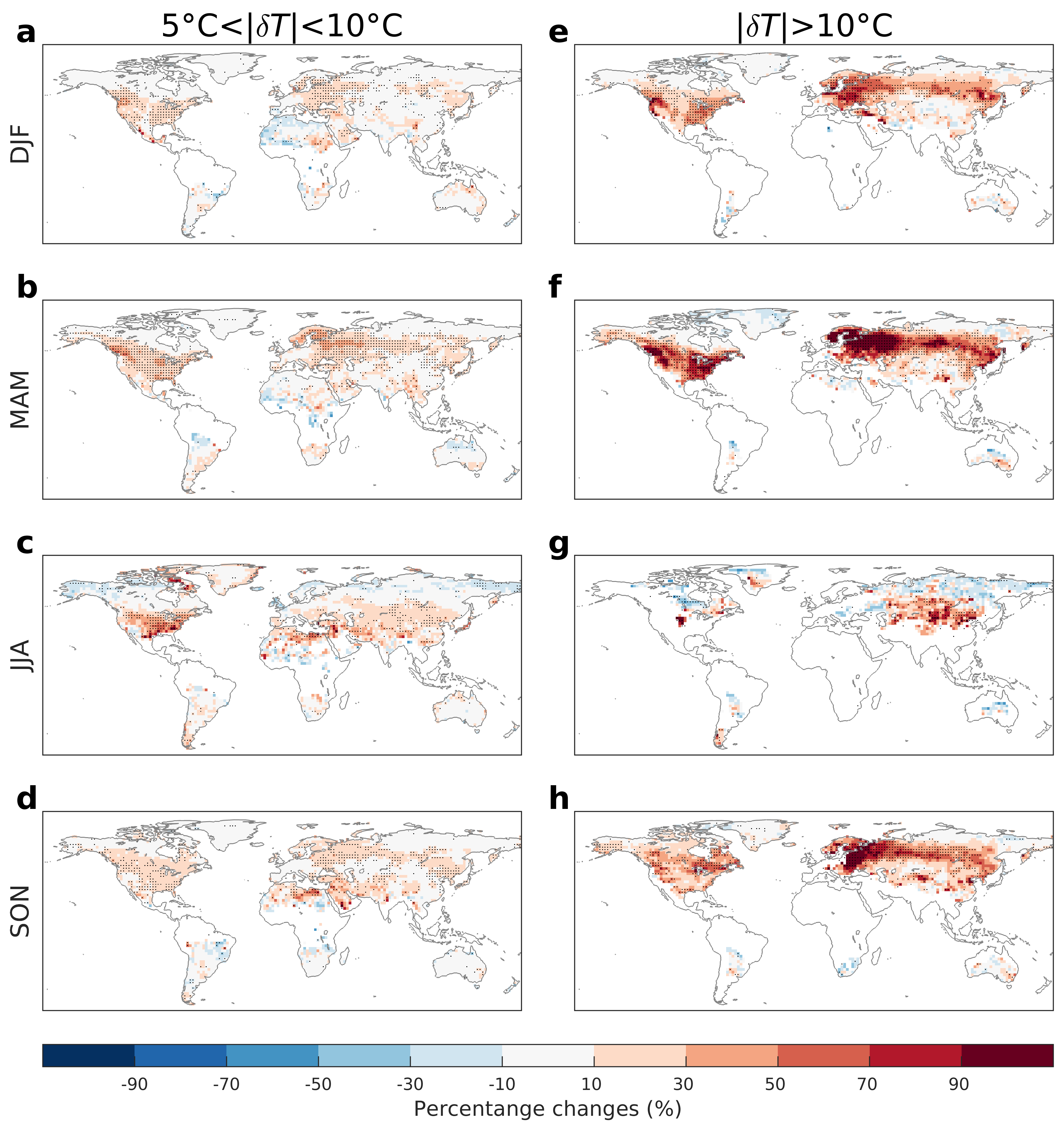


**Supplementary Fig. 6 The deforestation effect on the frequency of rapid warming or cooling events.** The percentage change ($\frac{deforest-globe - piControl}{\mathrm{piControl}}\times100\%$) in the frequency for temperature differences between neighboring days (δ*T*) within a given range: **a**, **b**, **c**, **d** 5 °C < |δ*T*| < 10 °C and **e**, **f**, **g** **h** |δ*T*| > 10 °C. **a**, **e**: DJF (December, January and February); **b**, **f**: MAM (March, April and May); **c**, **g**: JJA (June, July and August); **d**, **h**: SON (September, October and November).


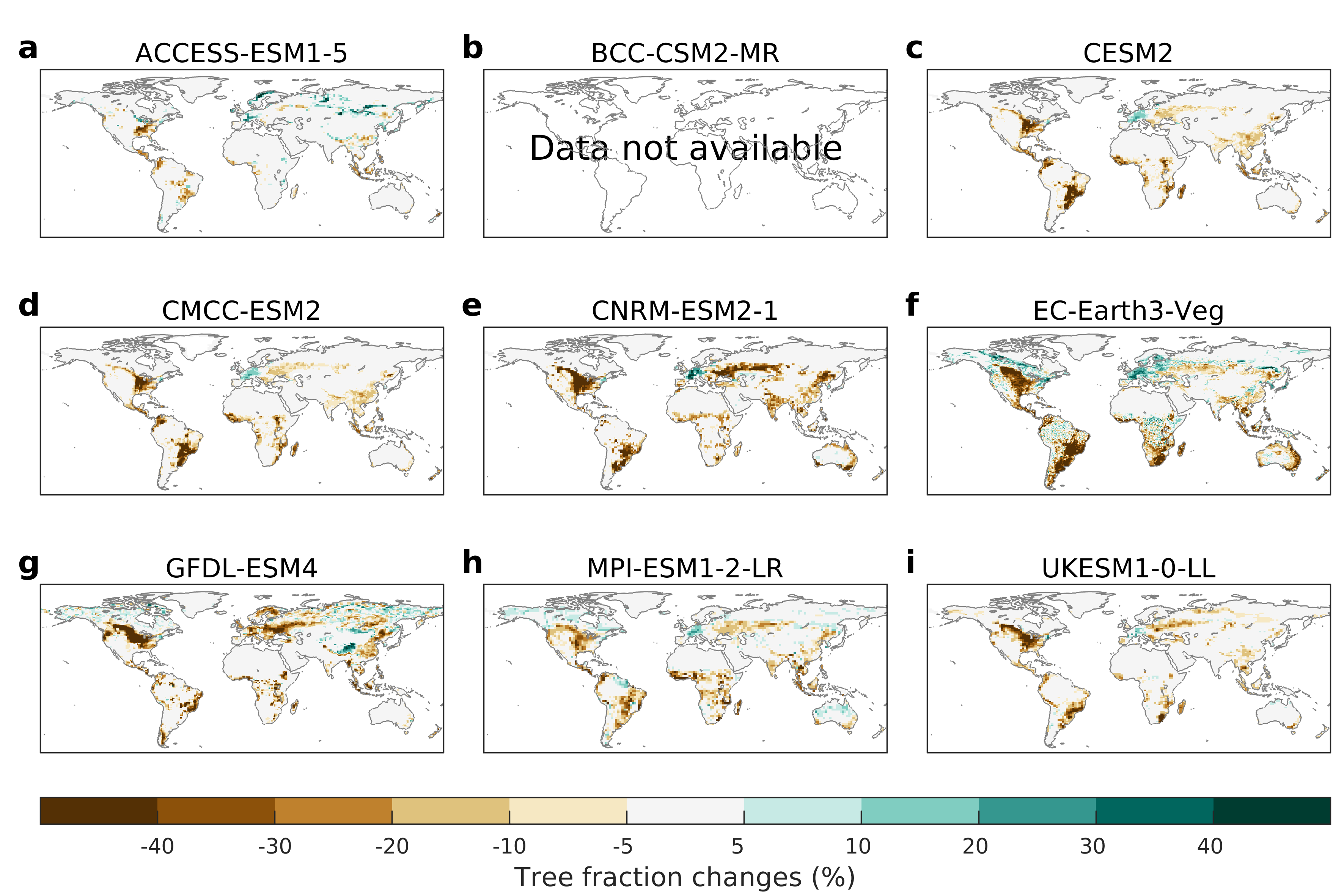


**Supplementary Fig. 7 Tree cover changes during the historical period.** Total tree fraction changes (historical minus hist-noLu) during the historical period (1850-2014) for each model. The data on the tree cover change for the BCC-CSM2-MR model are not available.


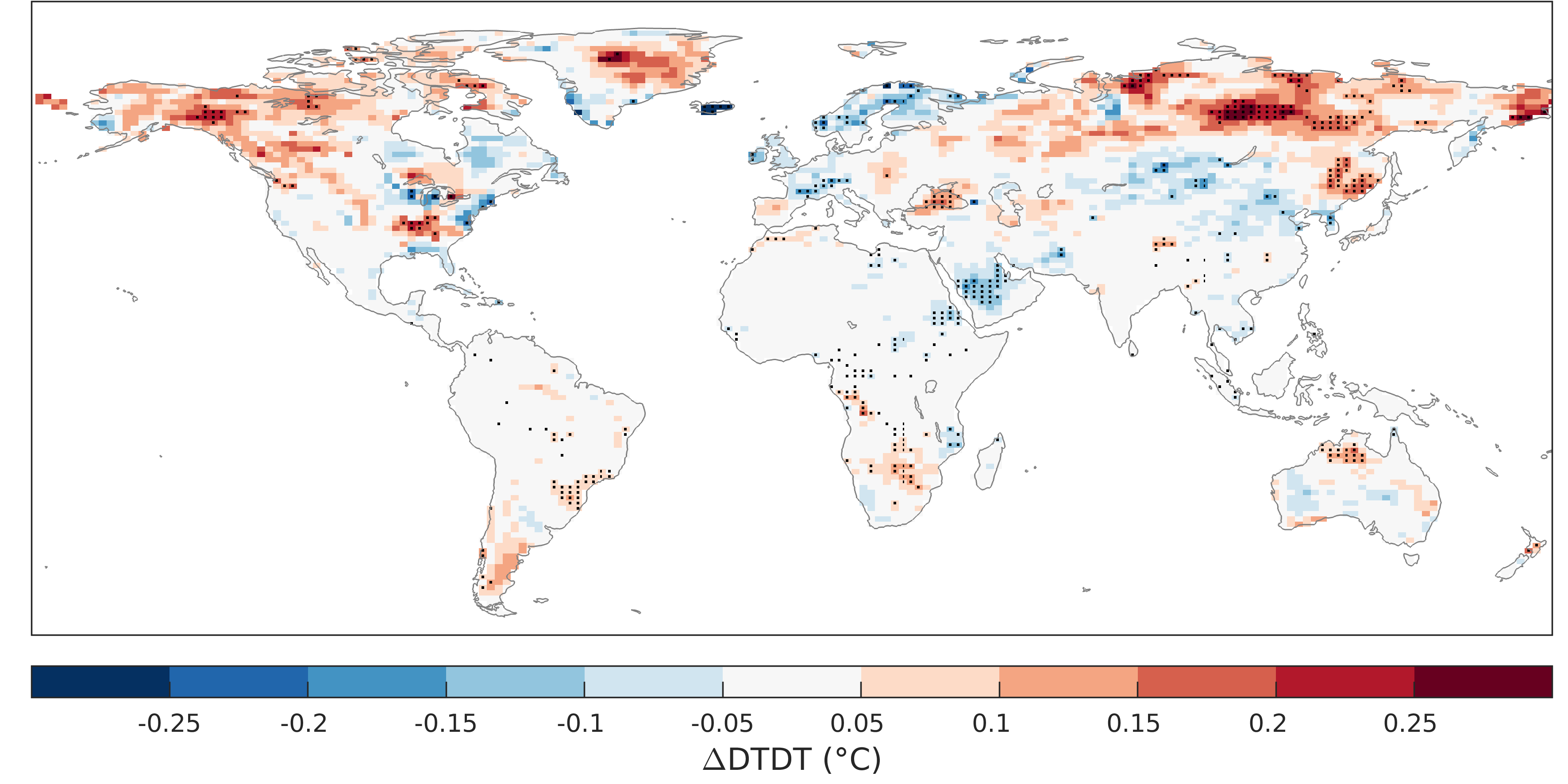


**Supplementary Fig. 8 The effect of historical tree cover changes on day-to-day temperature variability (DTDT) in DJF (December, January and February).** The DTDT change is from the ACCESS-ESM1-5 model, and the black dots indicate that the DTDT change is statistically significant at the 95% confidence level tested by Student’s *t*-test.

**References**

1. Ziehn, T. et al. The Australian Earth System Model: ACCESS-ESM1.5. *J. So. Hemisph. Earth.* **70**, 193-214 (2020).
2. Li, W. et al. Development of land surface model BCC_AVIM2.0 and its preliminary performance in LS3MIP/CMIP6. *J. Meteorol. Res.* **33,** 851-869 (2019).
3. Swart, N. C. et al. The Canadian Earth System Model version 5 (CanESM5.0.3). *Geosci. Model Dev.* **12**, 4823-4873 (2019).
4. Danabasoglu, G. et al. The Community Earth System Model Version 2 (CESM2). *J. Adv. Model. Earth Sy*. **12,** e2019MS001916 (2020).
5. Cherchi, A. et al. Global Mean Climate and Main Patterns of Variability in the CMCC-CM2 Coupled Model. *J. Adv. Model. Earth Sy.* **11**, 185-209 (2019).
6. Seferian, R. et al. Evaluation of CNRM Earth System Model, CNRM-ESM2-1: role of earth system processes in present-day and future climate. *J. Adv. Model. Earth Sy.* **11,** 4182-4227 (2019).
7. Doescher, R. et al. The EC-Earth3 Earth System Model for the Climate Model Intercomparison Project 6. *Geosci. Model Dev. Discussion.* (2021).
8. Dunne, J. P. et al. The GFDL Earth System Model version 4.1 (GFDL-ESM 4.1): Overall coupled model description and simulation characteristics. *J. Adv. Model Earth Sy.* **12** e2019MS002015 (2020).
9. Mauritsen, T. et al. Developments in the MPI-M Earth System Model version 1.2 (MPI-ESM 1.2) and its response to increasing CO 2. *J. Adv. Model. Earth Sy.* **11,** 998-1038 (2019).
10. Sellar, A. A. et al. UKESM1: Description and Evaluation of the UK Earth System Model. *J. Adv. Model. Earth Sy.* **11**, 4513-4558 (2019).
11. Duveiller, G., Hooker, J. & Cescatti, A. A dataset mapping the potential biophysical effects of vegetation cover change. *Sci. Data*. **5**, 180014 (2018).
